# Evaluation of Amlodipine and Imipramine Efficacy to Treat *Galleria mellonella* Infection by Biofilm-Producing and Antimicrobial-Resistant *Staphylococcus aureus*

**DOI:** 10.3390/antibiotics14020183

**Published:** 2025-02-11

**Authors:** Mariana Andrade, Joana Neves, Maria Bento, Joana Marques, Sofia G. Seabra, Henrique Silveira, Liliana Rodrigues, Ana Armada, Miguel Viveiros, Isabel Couto, Sofia Santos Costa

**Affiliations:** Global Health and Tropical Medicine, GHTM, Associated Laboratory in Translation and Innovation Towards Global Health, LA-REAL, Instituto de Higiene e Medicina Tropical, IHMT, Universidade NOVA de Lisboa, UNL, 1349-008 Lisbon, Portugal; a21001252@ihmt.unl.pt (M.A.); a21001575@ihmt.unl.pt (J.N.); a2022136@nms.unl.pt (M.B.); joana.a.marques@ihmt.unl.pt (J.M.); sgseabra@ihmt.unl.pt (S.G.S.); hsilveira@ihmt.unl.pt (H.S.); lrodrigues@ihmt.unl.pt (L.R.); aarmada@ihmt.unl.pt (A.A.); mviveiros@ihmt.unl.pt (M.V.); icouto@ihmt.unl.pt (I.C.)

**Keywords:** drug repurposing, *Staphylococcus aureus*, infection model, antimicrobial resistance, efflux, biofilm

## Abstract

**Background/Objectives:** Antimicrobial-resistant *Staphylococcus aureus* is a growing threat to human health for which alternative therapeutic options are needed. In this study, we aimed to evaluate the efficacy of amlodipine (AML) and imipramine (IMI) to treat *S. aureus* infection in the *Galleria mellonella* larval model by targeting efflux and biofilms, which are relevant contributors to antimicrobial resistance and virulence in *S. aureus*. **Methods:** In-house reared *G. mellonella* were used in virulence assays to determine the infective dose of two *S. aureus* strains differing in the expression of *norA* (gene encoding the native NorA efflux pump). Toxicology assays were conducted to determine the drugs’ LD_50_ for *G. mellonella*. Drug efficacy assays were performed to evaluate the potential of amlodipine, imipramine and the control drugs ciprofloxacin (CIP) and enalapril (ENA) to clear *S. aureus* infection in *G. mellonella*. **Results:** Survival analysis defined the infective dose as 1 × 10^7^ CFU/larva for both strains. High LD_50_ values were determined (CIP: >1000 mg/kg; AML: >640 mg/kg; IMI: 1141 mg/kg; ENA: >1280 mg/kg), revealing a high tolerance of *G. mellonella* to these drugs. AML at 15 mg/kg and IMI at 100 mg/kg increased the larvae survival by 20% (*p* = 0.04) and 11% (*p* = 0.11), respectively, also positively affecting health score indexes. In agreement with the literature, ciprofloxacin at >100 mg/kg promoted larvae survival by >73%. **Conclusions:** Amlodipine and imipramine show mild potential as new therapeutic options for managing *S. aureus* infections but are promising as new lead molecules. This study also reinforces *G. mellonella* as a sustainable, reliable model for drug evaluation.

## 1. Introduction

*Staphylococcus aureus* is one of the most important bacterial pathogens affecting human health, being part of the ESKAPE pathogens [1], and it is commonly associated with nosocomial infections [2]. It is the second cause of death attributable to and associated with bacterial antimicrobial resistance worldwide [3]. The occurrence of methicillin-resistant and multidrug-resistant phenotypes is frequent for this pathogen, limiting the available therapeutic options to treat infections caused by such strains. Consequently, the WHO has considered methicillin-resistant *S. aureus* (MRSA) a high-priority pathogen of public health importance for drug discovery to guide research, development and strategies to prevent and control antimicrobial resistance [4].

*S. aureus* has an array of antimicrobial resistance mechanisms and virulence factors that contribute greatly to the high burden of infections caused by this pathogen. Efflux is currently considered a pivotal mechanism of antimicrobial resistance in *S. aureus* [5] and other bacteria [6]. Several studies revealed efflux to be one of the first responses of bacteria when exposed to antimicrobials and other toxic compounds, allowing them to persist until a more stable resistance mechanism is established [7,8]. NorA is a main multidrug efflux pump in *S. aureus*, responsible for the extrusion of an array of substrates, including hydrophilic fluoroquinolones, quaternary ammonium compounds and dyes [5,9,10]. The overexpression of its encoding gene, *norA*, has been linked to the emergence of multidrug-resistance phenotypes [7,11].

Biofilm formation is a major mechanism contributing to the virulence of *S. aureus* and a common trait of *S. aureus* clinical strains [12]. Biofilm infections are often associated with increased resilience to antimicrobial chemotherapy and with traits of chronicity [13]. Biofilm formation in *S. aureus* is a complex process, involving several genetic factors contributing to adhesion, production of the extracellular matrix and regulation of the several steps of biofilm development [12]. In the past two decades, a link between efflux and bacterial biofilms has been hypothesized, and direct or indirect evidence has been gathered for many bacterial pathogens [14]. Efflux pumps may play a role in maintaining the structural integrity of biofilms by exporting components of the matrix, regulating biofilm formation and in quorum-sensing [14,15]. Studies in *S. aureus* have reported an overexpression of efflux pump genes, particularly *norA*, in biofilm-producing strains [16], supporting an efflux–biofilm relation in this bacterium.

Considering the urgent need for new therapeutic options to treat infections caused by drug-resistant *S. aureus*, we conducted an in silico drug repurposing study aiming to identify drugs that target both efflux and biofilms in *S. aureus* [17], unpublished data. In vitro testing of the candidate drugs identified two molecules, amlodipine and imipramine, that showed prominent efflux inhibitory and antibiofilm activities in *S. aureus*.

Amlodipine, a hydropyridine, is a calcium channel blocker prescribed in oral tablets, solutions or suspensions primarily to treat high blood pressure (hypertension) and certain types of chest pain (angina) [18]. Imipramine, a dibenzazepine, is a tricyclic antidepressant with a primary application in managing depression and anxiety disorders [18].

*Galleria mellonella* is an insect of the Lepidoptera order and Pyralidae family with a full metamorphosis cycle whose last larval stage has been increasingly used as a sustainable in vivo invertebrate model due to several advantages, including sharing similarities with innate immune responses of mammals [19]. The model may be used to study virulence properties of different bacterial [20,21,22] and fungal pathogens [23] and for antimicrobial efficacy testing [19,24,25], being a valuable tool to test new therapeutic options for the treatment of antimicrobial-resistant pathogens.

In this study, we evaluated the efficacy of amlodipine and imipramine to treat *S. aureus* infection in *G. mellonella*. The efficacy of ciprofloxacin was also studied as a control drug with data already available for *G. mellonella*. Enalapril was used as a negative control for which no efflux inhibitory activity was observed in the previous studies [17]. The drug efficacy assays were conducted using the ethidium bromide (EtBr)-adapted *S. aureus* ATCC 25923_EtBr strain [26], which presents increased efflux activity due to the overexpression of *norA* and is a strong biofilm producer.

## 2. Results

### 2.1. S. aureus Virulence and Infective Dose of Bacteria

**Figure 1:**
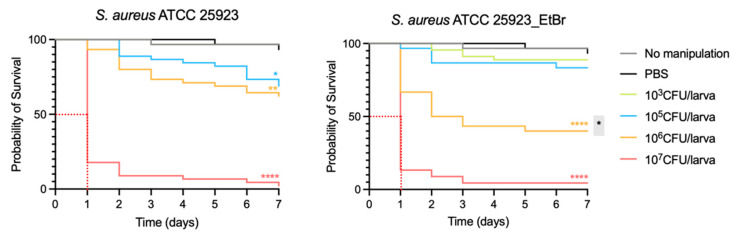
Kaplan-Meier survival plot of *G. mellonella* infected with *S. aureus* ATCC 25923 and *S. aureus* ATCC 25923_EtBr. The dotted red lines indicate the median survival time. Statistical differences were calculated against the PBS control and are highlighted as follows: * *p* < 0.05; ** *p* < 0.01; **** *p* < 0.0001. The black asterisk highlighted in gray indicates a statistically significant difference between the two strains at the 10^6^ CFU/larva inoculum. The results depicted are derived from three independent biological assays.

Analysis of the survival curves (Figure 1) revealed that the virulence of *S. aureus* isogenic strains is dose-dependent and consistent with previous assays with *S. aureus* [27,28]. Both strains exhibited greater virulence at the highest inoculum tested (10^7^ CFU/larva). When comparing the strains with the vehicle control (phosphate-buffered saline, PBS), for the parental *S. aureus* ATCC 25923 strain, the probability of survival was statistically significantly lower at 10^5^, 10^6^ and 10^7^ CFU/larva, with *p* < 0.05, *p* < 0.01 and *p* < 0.0001, respectively. For the *S. aureus* ATCC 25923_EtBr strain, the pattern was similar, yet there was no statistically significant effect on the probability of survival below 10^5^ CFU/larva inoculum. At 10^6^ CFU/larva, the probability of survival is notably lower (40% vs. 93.3% for vehicle control). When considering the strains’ virulence in relation to each other, both yielded the same median survival time (1 day). Yet, at 10^6^ CFU/larva, *S. aureus* ATCC 25923_EtBr, a *norA* overexpressing strain, is significantly (*p* < 0.05) more virulent than the parental strain, suggesting an increased virulence for this *S. aureus* strain.

The infective dose of bacteria is defined as the inoculum required to establish infection, usually corresponding to 60–80% mortality at 48 h [29]. Analysis of the survival curves revealed that the infective dose of bacteria was equivalent for both strains, corresponding to 1 × 10^7^ CFU/larva.

### 2.2. Drug Toxicity

**Figure 2:**
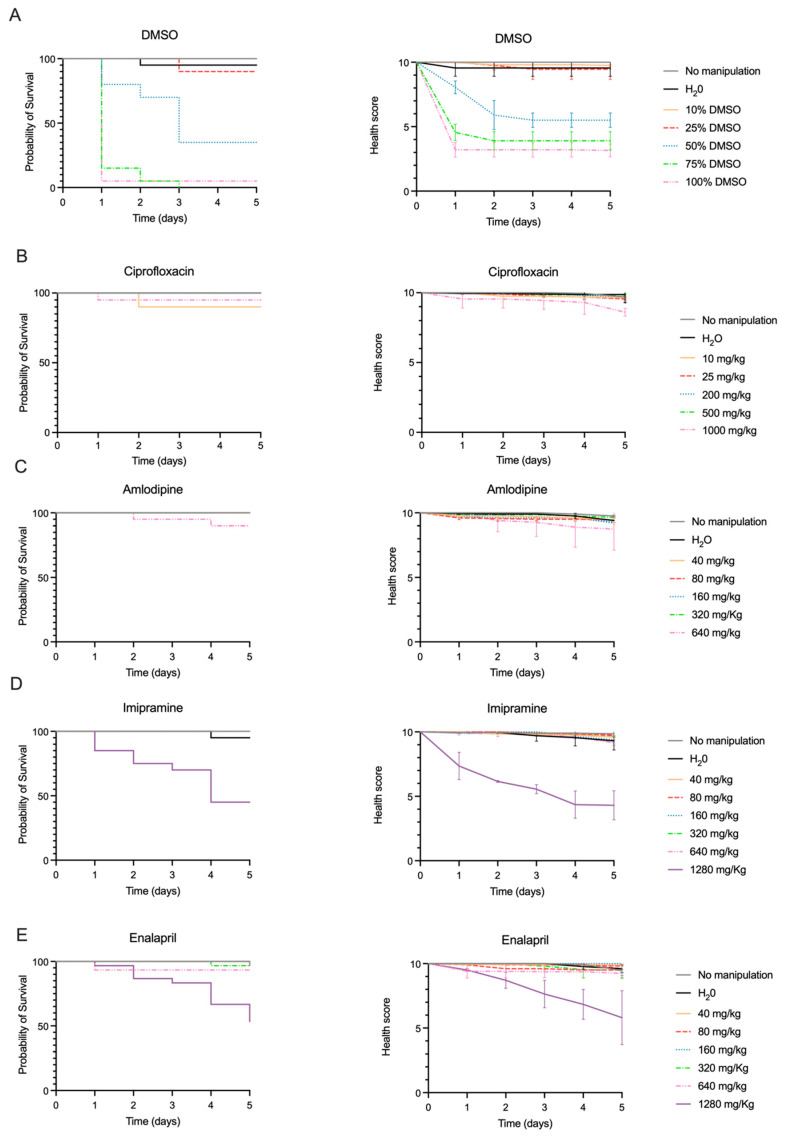
Kaplan-Meier survival plot (**left**) and health score indexes (**right**) of *G. mellonella* exposed to increasing doses (mg/kg or % *v*/*v*) of (**A**) DMSO, (**B**) ciprofloxacin, (**C**) amlodipine, (**D**) imipramine and (**E**) enalapril in toxicity assays. The results depicted correspond to three independent assays. For groups “No manipulation” and “H_2_O”, as well as for some drug doses, no effect on larvae survival and health score was observed, and therefore, the graph lines are superimposed.

Amlodipine, imipramine, enalapril and ciprofloxacin are drugs already approved for human use; therefore, their median lethal dose has already been established for rats and/or mice. Because their LD_50_ has not been established for *G. mellonella*, we carried out toxicity assays to ensure that the doses used in the efficacy assays were not toxic for this model. Additionally, since amlodipine was dissolved in dimethyl sulfoxide (DMSO), the LD_50_ of this solvent for *G. mellonella* was also determined. *G. mellonella* larvae showed good tolerability to DMSO, corresponding to an LD_50_ of 35% (*v*/*v*) for this solvent (Figure 2A). Based on this result, we established that all amlodipine solutions tested in *G. mellonella* should contain a maximum of 20% (*v*/*v*) DMSO to avoid affecting larvae survival and the health score index.

When analyzing the Kaplan-Meier survival curves and health score indexes (Figure 2B–E), we observed that *G. mellonella* had high tolerance to all four drugs, resulting in minimal or no mortality with consistent high health score index across most drug doses. Only imipramine at the highest dose tested (1280 mg/kg) caused a reduction of at least 50% in the probability of survival and in the health score index. Using the Spearman-Kärber method, we determined an LD_50_ for each of the four drugs in *G. mellonella* as follows: >1000 mg/kg for ciprofloxacin; >640 mg/kg for amlodipine; 1141 mg/kg for imipramine and >1280 mg/kg for enalapril.

### 2.3. Efficacy of Ciprofloxacin to Counteract G. mellonella Infection by S. aureus

**Figure 3:**
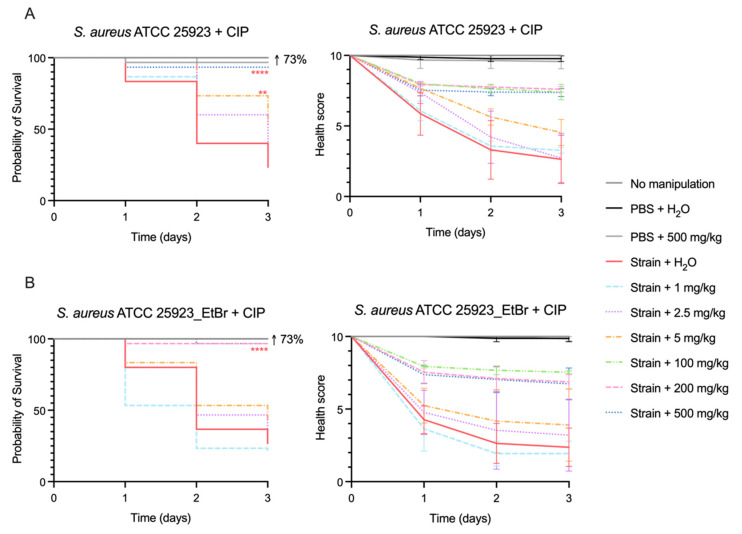
Kaplan-Meier survival plot (**left**) and health score indexes (**right**) of *G. mellonella* infected with *S. aureus* ATCC 25923 (**A**) and *S. aureus* ATCC 25923_EtBr (**B**) treated with increasing doses (mg/kg) of ciprofloxacin. Statistical differences were calculated against the control of non-treated larvae infected with the respective *S. aureus* strain and are highlighted as follows: ** *p* < 0.01; **** *p* < 0.0001. The black arrow indicates the increase in the probability of survival relatively to the infected and non-treated larvae. The results depicted are from three independent biological assays. For groups “No manipulation”, “PBS” and “PBS + 500 mg/kg CIP”, no effect on larvae survival and health score was observed, and therefore, the graph lines are superimposed.

We assessed the capacity of ciprofloxacin, an effluxable antimicrobial, to counteract *S. aureus* infection in *G. mellonella*. For these drug efficacy assays, we infected the larvae with the previously determined infective dose of bacteria (1 × 10^7^ CFU/larva) and then administered different ciprofloxacin doses. For both *S. aureus* strains, the response of *G. mellonella* to ciprofloxacin was dose-dependent with increasing probability of larvae survival as the ciprofloxacin dose increases (Figure 3). At concentrations below 5 mg/kg of ciprofloxacin, no effect was observed in larvae survival or health score. However, at 5 mg/kg, the probability of survival increased by 20% (*p* < 0.01) and 17% (not statistically significant) for *S. aureus* ATCC 25923 and *S. aureus* ATCC 25923_EtBr, respectively, when compared to the non-treated group. The ciprofloxacin treatment effect increased at doses higher or equal to 100 mg/kg with larvae survival probability rising by, at least, 73% (*p* < 0.0001). This effect was also observed in the health status of the larvae with improved health score indexes at the highest doses of ciprofloxacin (100, 200 and 500 mg/kg). At these high ciprofloxacin doses, the larvae remained fully motile but showed no recovery in their melanization status.

To determine the therapeutic dose of a drug, we considered the dose that yields a larvae mortality below 40% as defined by Ignasiak et al. [29], which for ciprofloxacin was 100 mg/kg for both strains. This value was lower than the therapeutic dose determined for ciprofloxacin (200 mg/kg) by Ignasiak et al. [29].

### 2.4. Efficacy of Amlodipine and Imipramine to Treat G. mellonella Infection by S. aureus

In a previous study [17], unpublished data, amlodipine and imipramine showed potential as efflux and biofilm inhibitors in vitro. Amlodipine (at 32 mg/L) and imipramine (at 64 mg/L) modulated the minimum inhibitory concentration (MIC) of the effluxable antimicrobial ciprofloxacin (CIP) by up to 8-fold and promoted intracellular EtBr accumulation (i.e., decrease efflux activity). They also presented the capacity, at sub-inhibitory concentrations, to inhibit biofilm growth by over 50% [17], unpublished data. Now, we assessed their in vivo efficacy in the *G. mellonella* infection model. These efficacy assays were carried out for *S. aureus* ATCC 25923_EtBr, the strain presenting increased efflux activity and strong biofilm production. As negative control, we assessed the efficacy of enalapril, an angiotensin-converting enzyme (ACE) inhibitor that did not show any in vitro efflux inhibitory activity in our previous study (i.e., no modulation of MICs of effluxable antimicrobials nor promotion of EtBr accumulation) [17], unpublished data. For the three drugs, the doses chosen to perform the efficacy assays were equivalent to the recommended dose for human use (AML: 0.15 mg/kg; IMI: 1.5 mg/kg; ENA: 0.6 mg/kg) plus ~10× and 100× equivalent to the recommended dose.

**Figure 4:**
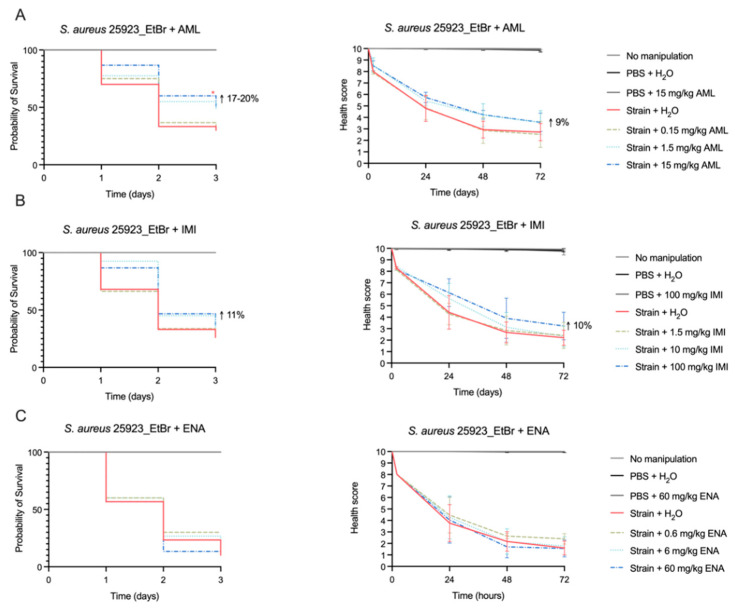
Kaplan-Meier survival plot and health score indexes of *G. mellonella* infected with *S. aureus* ATCC 25923_EtBr treated with (**A**) amlodipine, (**B**) imipramine and (**C**) enalapril. Statistical differences were calculated against the control of non-treated larvae infected with *S. aureus* ATCC 25923_EtBr and are highlighted as follows: * *p* < 0.05. The results depicted derive from at least three independent biological assays. The population (total number of larvae) analyzed varied between the conditions tested, ranging 30–60 for amlodipine/enalapril and 30–80 for imipramine. For groups “No manipulation”, “PBS” and “PBS + highest drug dose”, no effect on larvae survival and health score was observed, and therefore the graph lines are superimposed.

When analyzing the Kaplan-Meier survival curves and health score indexes (Figure 4), a mild effect of both amlodipine and imipramine on larvae survival and health status was noted. We observed increased larvae probability of survival at the highest doses tested. At 1.5 mg/kg, although not statistically significant, this parameter increased by 17% (*p* = 0.08), whereas a statistically significant effect of 20% (*p* = 0.04) was observed with 15 mg/kg of amlodipine. Regarding the health score index, it was increased by 9% for both 1.5 and 15 mg/kg of amlodipine (not statistically significant). For the lowest drug dose, no effect was observed. For imipramine, an 11% (*p* = 0.11) increase in the probability of survival was detected, albeit not statistically significant, at the highest dose tested of 100 mg/kg. This was also reflected in the health score index, which improved by 10% at 100 mg/kg. As expected, enalapril, did not show any positive effect in larvae survival or health scores (Figure 4C).

## 3. Discussion

*Staphylococcus aureus* is amongst the most relevant human pathogens, causing a wide range of mild to life-threatening infections [30]. The emergence of methicillin-resistant and multidrug-resistant strains has become an increasing challenge in healthcare settings, which along with the scarce discovery of new antimicrobials indicates the urgent need to develop alternative therapeutic strategies. Efflux pumps and biofilm formation are important mechanisms contributing to *S. aureus* antimicrobial resistance and virulence and thus promising targets for the development of new drugs. In particular, their potential interplay underscores the relevance of identifying drugs capable of targeting both mechanisms. Previous studies showed that known NorA inhibitors can reduce biofilm formation in *S. aureus* and *S. epidermidis* strains [16,31]. This suggests that targeting efflux activity could be a promising approach to combat *S. aureus* infections caused by both antimicrobial-resistant and biofilm-producing strains.

Drug repurposing, defined as identifying new applications for drugs already approved for clinical use, has been pointed out as a valuable approach to speed up drug discovery, saving valuable resources and time, by advancing directly to pre-clinical testing [32]. This approach has been successfully applied, as exemplified by the potential for repurposing of the antihelmintic drug niclosamide as an inhibitor of quorum-sensing in *Pseudomonas aeruginosa*, reducing *P. aeruginosa* pathogenicity in an insect model [33].

Our group has recently conducted an in silico drug repurposing study to identify candidate drugs dually targeting staphylococcal efflux and biofilms. The in vitro testing of over 50 representative candidate drugs revealed amlodipine and imipramine amongst the most promising drugs inhibiting both efflux activity and biofilm formation in *S. aureus* biofilm-producing strains that showed also increased efflux activity [17]. Our next step was to assess their activity against the same set of *S. aureus* strains in vivo. For that, we used the *G. mellonella* infection model, available in our laboratory, with optimized rearing and infection protocols.

To validate the efficacy of drug candidates for repurposing, we optimized multiple parameters to perform efficacy assays in *G. mellonella*. The first parameter to be determined was the infective dose of bacteria, corresponding to an appropriate bacterial inoculum that allowed the infection to be established in the larva without promoting excessive mortality [29]. By performing virulence assays, we were able to determine that the infective dose of bacteria was 1 × 10^7^ CFU/larva for both strains studied. The virulence assays also allowed the comparison of virulence potential between *S. aureus* ATCC 25923 and *S. aureus* ATCC 25923_EtBr (derivative strain overexpressing the gene encoding the NorA efflux pump). The behavior of the strains was similar, showing that the virulence was proportional to the bacterial inoculum; i.e., higher inoculum levels result in more pronounced virulence. However, at 10^6^ CFU/larva, a statistically significant difference was detected between the strains, with the *norA* overexpressing strain being more virulent, supporting the hypothesis that efflux may play an important role in *S. aureus* virulence.

*G. mellonella* is described as a versatile in vivo model that can be used in drug discovery studies for many reasons. One of them is the high tolerability to different solvents when compared to cell lines or other animal models [24]. Low water solubility was a common trait for most of the drugs studied, including amlodipine. Its solubility increases in organic solvents, such as DMSO, used in this work. The toxicity assays performed in this study established an LD_50_ of 35% for this solvent, confirming the high tolerability of *G. mellonella* in accordance with previous reports [34].

Ciprofloxacin, amlodipine, imipramine and enalapril are already approved for human use; therefore, their toxicity is already known. In our work, we determined a ciprofloxacin LD_50_ for *G. mellonella* of >1000 mg/kg, which is in agreement with the findings of Ignasiak and colleagues [29] (ciprofloxacin LD_50_ of >2000 mg/kg). The *G. mellonella* LD_50_ value falls within the range of the value previously determined for rat (>2000 mg/kg), attesting a similar tolerance of *G. mellonella* and mammals to this hydrophilic fluoroquinolone. To the best of our knowledge, this study is the first to report LD_50_ values of amlodipine (>640 mg/kg), imipramine (1141 mg/kg) and enalapril (>1280 mg/kg) for *G. mellonella*. These high values indicate a higher tolerability for these drugs by this invertebrate compared to murine and rat models for which oral LD_50_ values for amlodipine were 37 mg/kg and 393 mg/kg, respectively. For imipramine, the oral LD_50_ values were 188 mg/kg and 250 mg/kg, respectively, and for enalapril, the oral LD50 for rats was 2973 mg/kg [18]. These results support *G. mellonella* as a valuable tool for toxicity studies.

Ciprofloxacin is a hydrophilic fluoroquinolone used for the treatment of several bacterial infections [35] that inhibits the bacterial DNA gyrase and topoisomerase IV [36]. Ciprofloxacin was included in this study as a control drug, with toxicity and drug efficacy data already available in the literature, and also because it is a substrate of many multidrug efflux pumps, including NorA [5]. The recommended therapeutic oral dose for ciprofloxacin is 20 mg/kg/day for children and 10–15 mg/kg (500 mg/twice/day) for adults [18]. We found that at a 100 mg/kg dose, ciprofloxacin was effective in clearing *S. aureus* infection in *G. mellonella*, which is a value slightly lower than the one reported by Ignasiak et al. (200 mg/kg) [29], but it is still ~10× higher than the one recommended for human use. According to the FDA, the recommended therapeutic dose of amlodipine and imipramine for adults is 5–10 mg/day and 50–150 mg/day, respectively. The dose range tested in this work comprehends the recommended dose for human use (considering an adult of 65 kg) and doses approximately 10 and 100 times higher [18]. The lowest drug doses tested, which mimic the recommended human doses of amlodipine and imipramine, did not present any activity against *S. aureus* infection in *G. mellonella*. However, the highest dose tested of amlodipine (15 mg/kg) showed a statistically significant effect promoting larvae survival by 20%. Imipramine had a milder effect at 100 mg/kg, increasing the probability of larvae survival by 11%. As a negative control, we used enalapril, which is a drug that in our earlier study did not show efflux inhibitory activity in vitro. As expected, this ACE inhibitor, used to treat high blood pressure and congestive heart failure [18], did not present any activity in treating infection in *G. mellonella* caused by a *S. aureus* strain with increased efflux activity and strong biofilm production.

In mice and rats, mortality was observed at single oral doses of amlodipine maleate equivalent to 40 mg/kg and 100 mg/kg, respectively [18]. In humans, an overdose of amlodipine can lead to several health problems including a high degree of peripheral vasodilatation. Several fatal cases of amlodipine overdose have been reported associated with blood concentrations as low as 0.95 mg/L [37]. For imipramine, the human range of toxicity includes single doses greater than 5 mg/kg [18]. An overdose of imipramine can affect the cardiovascular or neurological systems with fatal cases associated with doses ranging from 10 to 210 mg/kg [38]. These data indicate that the doses that showed an effect in the *G. mellonella* infection model will result in toxicity to humans, impairing their direct use for the treatment of *S. aureus* infection. Nonetheless, our results show that these drugs have the potential to be used as lead compounds to design new and improved molecules with higher efficacy and lower toxicity to humans, enabling their future application in the treatment of *S. aureus* infection. We have previously detected a synergic effect between amlodipine/imipramine and ciprofloxacin in in vitro assays [17], unpublished data. Some studies have also reported synergism between amlodipine and beta-lactams (oxacillin) in *S. aureus* [39] or between imipramine and moxifloxacin in *M. tuberculosis* [40]. These data together with the capacity to inhibit efflux in *S. aureus* indicate that further studies should focus on exploring the potential of both drugs to act as adjuvants of ciprofloxacin in the *G. mellonella* infection model. Their antibiofilm properties could also be explored in a biofilm infection model using this invertebrate model.

In this study, *G. mellonella* has proven to be a sustainable alternative infection model for evaluating new therapeutic options against infections caused by *S. aureus*. Other studies have also employed this approach to evaluate drug efficacy in *S. aureus* and other bacteria [25,41,42]. Nevertheless, because this is an invertebrate animal model, some limitations have to be taken into consideration. *G. mellonella* lacks an adaptative immune response, which limits the mimicking of human immune responses, thus hindering the direct extrapolation of results [43]. Due to the invertebrate nature of this model, the biology and physiopathology of disease are different from those in humans and consequently affect the pharmacokinetics of drugs [19]. All these differences may explain the need for the high-efficacy doses reported in this work to treat *S. aureus* infection. To further validate these findings, efficacy studies can be extended to more complex animal models, such as mice, to complement the insights gained from the *G. mellonella* infection model.

## 4. Materials and Methods

### 4.1. Bacterial Strains, Antimicrobials and Candidate Drugs

In this work, we used the fully susceptible strain *S. aureus* ATCC^®^ 25923™ and its derivative *S. aureus* ATCC 25923_EtBr, which overexpresses the *norA* efflux pump gene following an EtBr adaptation process [26]. The strains were kept at −80 °C in tryptone soya broth (TSB, Oxoid Ltd., Hants, UK) supplemented with glycerol at 10% (*v*/*v*). Prior to their use, the strains were transferred to tryptone soya agar (TSA, Oxoid Ltd., Hants, UK) and grown at 37 °C overnight. The EtBr-adapted strain was grown in TSA supplemented with EtBr at 32 mg/L.

Ciprofloxacin (CIP, Sigma-Aldrich, Steinheim, Germany), amlodipine (AML, Sigma-Aldrich, Steinheim, Germany), imipramine (IMI) and enalapril (ENA) (Tokyo Chemical Industry, Tokyo, Japan) were acquired in powder form. Stock solutions of CIP, IMI and ENA were prepared in demineralized water, whereas stock solutions of AML were prepared in DMSO. All dilutions were prepared in demineralized water.

### 4.2. Previous in Silico Drug Repurposing Study

We have previously conducted an in silico drug repurposing study in which a list of potential targets, comprising all *S. aureus* membrane transporters and biofilm-associated proteins, was used to interrogate the DrugBank database and compile a list of drugs targeting homologs of these proteins. A subset of candidate drugs identified as potential efflux inhibitors and/or antibiofilm agents was tested in vitro against *S. aureus* ATCC 25923 and ATCC 25923_EtBr strains. Candidate drugs were assessed for their ability to reduce the MICs of effluxable antimicrobials (including CIP) and evaluating ethidium bromide accumulation by fluorometry [7,44]. Drugs showing a significant effect in both assays were considered as presenting efflux inhibitory activity and were further tested for their potential to inhibit biofilm formation using the crystal violet adhesion method.

### 4.3. Galleria mellonella Colony

*G. mellonella* were reared and maintained at GHTM/IHMT-NOVA at 28 °C in the dark, under a high-nutrition diet, modified from Jorjão et al. [45], consisting of corn flour, dried yeast, soy flour, dry milk, honey, glycerol and beeswax blocks, acquired from local commerce sources. Larvae at the final instar stage, weighing 400 ± 50 mg, fully motile and with no signs of melanization were selected for the infection assays.

### 4.4. Determination of the Infective Dose of Bacteria

The infective dose of both *S. aureus* strains was assessed as described previously in Andrade et al. [27] and Garrine et al. [28]. Briefly, bacteria were incubated in TSB at 37 °C and 180 rpm overnight. The cells were collected and washed in PBS (NZYTech, Lisbon, Portugal) and then adjusted to ~5 × 10^8^ CFU/mL to achieve the highest inoculum, corresponding to 1 × 10^7^ CFU/larva. The cell suspension was then diluted in PBS to obtain 5 × 10^6^ CFU/mL and 5 × 10^4^ CFU/mL, corresponding to 1 × 10^5^ CFU/larva and 1 × 10^3^ CFU/larva, respectively. Bacteria enumeration was determined by CFU counting.

For the infection assays, groups of ten *G. mellonella* larvae were kept in 12-well plates in the dark at 37 °C. Each assay included five different groups: (i) no manipulation; (ii) PBS; (iii) 10^3^ CFU/larva; (iv) 10^5^ CFU/larva and (v) 10^7^ CFU/larva. All larvae were inoculated with 20 μL of each inoculum or PBS by injection with a Hamilton syringe (ITO Corporation, Fuji, Japan) in the last right proleg. Larvae survival was monitored each 24 h post-infection for seven days. At least three independent infection assays were performed for each strain. The infective dose of bacteria was considered as the one that caused 60–80% mortality in 48 h but not 100% in 24 h [29].

### 4.5. Evaluation of Drug Toxicity in G. mellonella

The toxicity of ciprofloxacin, amlodipine, imipramine and enalapril in *G. mellonella* was determined, as well as that of DMSO, which is the solvent of amlodipine. Different ranges of drug doses were tested as follows: 10, 25, 200, 500 and 1000 mg/kg for ciprofloxacin; 40, 80, 160, 320 and 640 mg/kg for amlodipine; and 40, 80, 160, 320, 640 and 1280 mg/kg for imipramine and enalapril. The toxicity of DMSO was tested at 10, 25, 50, 75 and 100% (*v*/*v*).

The drug toxicity assays included two control groups (no manipulation and inoculation with sterile H_2_O) and a group for each drug concentration. The assays were conducted as indicated in Section 4.2. For these assays, 10 μL of each drug/solvent dose were inoculated in the last left proleg by injection with a Hamilton syringe. Larvae survival was monitored each 24 h post-drug administration for five days. We also monitored the larvae health status, using a modified health score index (Table 1) proposed in previous studies [46,47].

At least three independent drug toxicity assays were performed. The LD_50_ of each drug for *G. mellonella* was determined using the Spearman-Kärber method [48], following the formula below:

$$logLD50=\frac{log\;\left(highest\;dose\;giving\;100\%\;deaths\right)+0.5\times (total\;number\;of\;deaths)}{number\;of\;total\;larvae\;per\;dose}$$

### 4.6. Drug Efficacy Assays in G. mellonella

The efficacy of the drug candidate for repurposing amlodipine and imipramine in treating infection by *S. aureus* was tested in *G. mellonella*. Additionally, we used as positive and negative controls, respectively, ciprofloxacin, an effluxable antibiotic for which efficacy data in *G. mellonella* are available, and enalapril, an FDA-approved drug with no efflux inhibitory activity detected in the in vitro studies [29]. The infection assays were only conducted for strain *S. aureus* ATCC 25923_EtBr, which has the traits of interest (increased efflux activity and strong biofilm production).

The drugs’ doses for the efficacy assays were determined considering the recommended human doses (AML: 10 mg/day; IMI: 100 mg/day; ENA: 40 mg/day) and an adult with an average weight of 65 kg. Thus, three doses were tested: (i) equivalent to the recommended human dose (AML: 0.15 mg/kg; IMI: 1.5 mg/kg; ENA: 0.6 mg/kg); (ii) 10× and (iii) 100× the equivalent of the recommended dose.

Groups of ten larvae were inoculated with the infective dose bacteria as described in Section 4.2, and after 2 h, larvae were treated with 10 μL of different doses of amlodipine (0.15, 1.5 and 15 mg/kg), imipramine (1.5, 10 and 100 mg/kg), enalapril (0.6, 6 and 60 mg/kg) or ciprofloxacin (2.5, 5, 100, 200 and 500 mg/kg). Control groups were also included in these assays: namely, no manipulation, PBS + H_2_O, PBS + highest drug dose tested.

Larvae survival and health scores were monitored each 24 h post-infection for three days. At least three independent drug efficacy assays were performed.

### 4.7. Data Analysis

Statistical analysis was performed using GraphPad Prism v 8.0.1 (San Diego, CA, USA). Kaplan-Meier survival curves and the median survival time were determined, and survival rates were compared using the log-rank (Mantel-Cox) test. The significance level considered was α = 0.05.

## 5. Conclusions

In this study, we demonstrate that amlodipine, an antihypertensive drug, and to a lesser extent imipramine, an antidepressant, hold potential as lead compounds for developing new treatments for *S. aureus* infections. These drugs could be optimized to enhance their activity or evaluated as adjuvants to efflux-prone antibiotics such as ciprofloxacin. Additionally, this work highlights the utility of *G. mellonella* as a cost-effective infection model for rapidly assessing drug efficacy, thereby accelerating the drug discovery process.

## Figures and Tables

**Table 1:** Modified health score index used in this study.

Category	Description	Score
Activity	No activity	0
	Minimal activity on stimulation	1
	Active when stimulated	2
	Active without stimulation	3
Cocoon formation	No cocoon	1
	Partial cocoon	0.5
	Full cocoon	0
Melanization	Complete melanization (black)	0
	Dark spots on brown larva	0
	≥3 spots/segments on beige larva	2
	<3 spots/segments on beige larva	3
	No melanization	4
Survival	Dead	0
	Alive	2

## Data Availability

The original contributions presented in this study are included in the article. Further inquiries can be directed to the corresponding author.

## Abbreviations

The following abbreviations are used in this manuscript:

MDPI	Multidisciplinary Digital Publishing Institute
DOAJ	directory of open access journals
AML	amlodipine
CIP	ciprofloxacin
DMSO	dimethyl sulfoxide
ENA	enalapril
EtBr	ethidium bromide
IMI	imipramine
PBS	phosphate-buffered saline
